# Supplementary material for: Characteristics and conflicts of interest at Food and Drug Administration Gastrointestinal Drug Advisory Committee meetings
Source: PLoS One. 2021 May 26;16(5):e0252155. doi: 10.1371/journal.pone.0252155 (PMC8153474; doi:10.1371/journal.pone.0252155)
Supplement: Attachment — Submitted filename: Response to reviewers.pdf [file pone.0252155.s003.pdf]

St. Michael's
Inspired Care.
Inspiring Science.

Samir C. Grover, MD, MEd, FRCPC
Division of Gastroenterology, St. Michael's Hospital
Associate Professor, Department of Medicine, University of Toronto

February 23 2021
Dr. Gentili
Academic Editor, PLOS ONE

Dear Dr. Gentili and the members of the editorial board:

Thank you for considering and giving us the opportunity to revise our manuscript titled "Characteristics and Conflicts of Interest at Food and Drug Administration Gastrointestinal Drug Advisory Committee Meetings" (PONE-D-20-36649). We would also like to thank the reviewers for their comments. Our responses are below.

EDITOR

1. *Please ensure that your manuscript meets PLOS ONE's style requirements, including those for file naming.*
    a. We have made the required formatting changes.

2. *We note that you have indicated that data from this study are available upon request. PLOS only allows data to be available upon request if there are legal or ethical restrictions on sharing data publicly*
    a. We have included the data as supplementary file 1.

3. *Please confirm that this does not alter your adherence to all PLOS ONE policies on sharing data and materials, by including the following statement: "This does not alter our adherence to PLOS ONE policies on sharing data and materials." (as detailed online in our guide for authors. Please include your updated Competing Interests statement in your cover letter; we will change the online submission form on your behalf.*
    a. We have included this statement in our cover page.

REVIEWER 1

4. *I would suggest that the authors look at the number of COI waivers per meeting to see what percent of the people had a level of conflicts than would normally be allowed and how those people voted? Similarly, given the influence that committee chairs can have over the process, it would be interesting to look at whether the chairs had a COI and how they voted.*
    a. We have stated that all voting members with COI had waivers, and that ten of the chairs had COI. Both of these statements are in the *Results* section, *Conflicts of interest* subsection.

5. *Were there any committee meetings where a majority of the members had a conflict?*
    a. There were no meetings where a majority of the members had a conflict.

16-036 Cardinal Carter Wing, 30 Bond Street, Toronto, ON., M5B 1W8
Office: (416) 864-5628 Fax: (416) 864-5882

6. *The authors should describe what level of COI disqualifies someone from sitting on a committee and give more detail about how those criteria changed with the 2002 revision of the disclosure guidelines and the 2007 FDA amendments.*
    a. We have added several details regarding this legislature in the section "Discussion".

7. *Lines 187-189: I'd like to see a bit more detailed analysis of these numbers, i.e., out of x instances where the advisory committee recommended approval, the FDA approved y but did not approve z. Out of x studies where the committee did not recommend approval the FDA did not approve y but approved z. This could be done in the form of a 2x2 table.*
    a. We have added in a table as suggested, which is now table 2.

8. *How were discrepancies between people who extracted data resolved?*
    a. We have stated that discrepancies were resolved by consensus in the *Methods* section, *Data collection* subsection.

9. *Line 157: What was the basis for dichotomizing sample size between <500 and ≥500. Also, I think that for the number of clinical trials the authors mean 1 vs >1, not 1 vs 12.*
    a. We agree that the dichotomization of certain variables could have introduced bias into our analysis, and have listed this as a limitation in the *Discussion* section.

10. *In the Methods under the Sample subheading the authors said that they also looked at votes for whether a medication had demonstrated safety and efficacy and whether a medication had a favorable risk-benefit profile. Were there no votes on these two questions at any of the committee meetings?*
    a. Meeting members voted on either of the above questions. In the 34 meetings we examined, there were no meetings in which both sets of questions were answered

11. *Why didn't the authors use multiple linear regression to examine whether the various factors that they analyzed determined a negative versus positive vote?*
    a. We did not use multiple regression to avoid overfitting this small data set. We have stated this in the *Methods* section, *Analysis* subsection.

REVIEWER 2

12. *My major concern regards data sharing. Indeed, the GIDAC meetings are public, so the raw data are accessible. However, to ensure reproducibility, given that the authors made several coding and extraction decisions, curated datasets of all extracted information from the GIDAC meetings for each application and indication should be included, along with all extracted information about the individual voting members. Synthetically, the readers need to have access to the entirety of the data supporting their findings and be able to re-run the analyses, without necessarily having to extract everything ab novo. This information should*

be included as supplementary material, for example in several tables, or deposited in a public repository such as the Open Science Framework.

   a. We have included the data as a supplementary file.

13. *Another concern is the rationale for dichotomizing all predictor variables, particularly continuous ones like sample size? There are many problems with dichotomization in general, see for example*[1]2/23/2021 1:35:00 AM
    a. We acknowledge the deficiencies with dichotomizing predictor variables. However, given the relatively small sample size and the likely lack of a normal distribution for the continuous variables, we felt it would be more appropriate to dichotomize the variables. Additionally, we based our dichotomization partially on a similar analysis on oncology drug advisory meetings by Tibau et al. (2016). We have added in statement regarding the limitation on data dichotomization in the *Discussion*, limitations paragraph.

14. Also, the statistical significance threshold should be corrected for the multiple comparisons performed.
    a. We have added in a Bonferroni correction in the *Methods* section.

Thank you once again for your consideration of this manuscript. We look forward to your review and comments.

Yours sincerely,

Samir C. Grover, MD, MEd, FRCPC
Division of Gastroenterology
St. Michael's Hospital
